# Supplementary material for: Predicting the intention to adopt wearable payment devices in China: The use of hybrid SEM-Neural network approach
Source: PLoS One. 2022 Aug 30;17(8):e0273849. doi: 10.1371/journal.pone.0273849 (PMC9426926; doi:10.1371/journal.pone.0273849)
Supplement: S1 Appendix — (DOCX) [file pone.0273849.s001.docx]

**APPENDIX 1.** Survey Instrument

| **Code** | **Items** |
| --- | --- |
| PE-1 | Wearable payment device is a useful tool for conducting transactions |
| PE-2 | Wearable payment devices enable me to conduct transactions easily |
| PE-3 | I can conduct transactions faster on wearable payment devices as compared to cash transactions |
| PE-4 | I find wearable payment devices useful in my daily life |
| PE-5 | Using wearable payment devices improves the quality of my daily life |
| PE-6 | Using wearable payment devices helps accomplish things more quickly |
| EE-1 | It would be easy for me to understand the operation of wearable payment devices |
| EE-2 | It is easy for me to become skillful at using wearable payment devices |
| EE-3 | I conduct transactions through mobile devices because many people are doing so |
| EE-4 | Learning how to use wearable payment devices is easy for me |
| EE-5 | I find conducting transactions through devices is convenient for me |
| SI-1 | People who are important to me think that I must use wearable payment devices |
| SI-2 | People who influence my behavior think that I should use wearable payment devices |
| SI-3 | People in my society who use wearable payment devices have more prestigious than those who do not |
| SI-4 | People whose opinions are valuable to me would prefer that I use wearable payment devices |
| SI-5 | People whose opinions I valued preferred that I use this wearable payment devices |
| FC-1 | I have the necessary knowledge to use wearable payment devices |
| FC-2 | Wearable payment devices are compatible with other devices that I use |
| FC-3 | I have the resources necessary to use wearable payment devices |
| FC-4 | Guidance will be available to me in the use of wearable payment devices |
| FC-5 | A specific person (or group) is available for assistance with the difficulties |
| HM-1 | Using wearable payment devices is fun |
| HM-2 | Using wearable payment devices is enjoyable |
| HM-3 | Using wearable payment devices is exciting |
| HM-4 | Using wearable payment devices is delightful |
| HM-5 | Using wearable payment devices is entertaining |
| LC-1 | Using wearable payment devices is compatible with all aspects of my lifestyle |
| LC-2 | Using wearable payment devices is completely compatible with my current situation |
| LC-3 | Using wearable payment devices fits into my lifestyle |
| LC-4 | Using wearable payment services fits well with the way I like to make the bills payment |
| LC-5 | Using wearable payment services instead of alternative modes of payment (e.g. credit card and cash) |
| PT-1 | I believe wearable payment is trustworthy |
| PT-2 | I believe wearable payment keeps customers' best interests in mind |
| PT-3 | I believe wearable payment keeps its promises and commitments |
| PT-4 | I believe wearable payment is reliable |
| PT-5 | Trust and enjoyment become the dominant drivers of wearable payment that offset the risk perceptions |
| IWPD-1 | I intend to use wearable technology at every opportunity in the future |
| IWPD-2 | I plan to increase my use of wearable technology in the future |
| IWPD-3 | It is likely that I will continue using wearable payment devices in future |
| IWPD-4 | Given that I have a smartphone capable of accessing internet, I will continue using wearable payment devices |
| IWPD-5 | I will continue using wearable payment systems in future |
| IWPD-6 | I intend to use wearable devices for planning my payment activities |
