# Supplementary material for: Predicting the intention to adopt wearable payment devices in China: The use of hybrid SEM-Neural network approach
Source: PLoS One. 2022 Aug 30;17(8):e0273849. doi: 10.1371/journal.pone.0273849 (PMC9426926; doi:10.1371/journal.pone.0273849)
Supplement: Attachment — Submitted filename: Reply to reviewer (PONE-D-22-02148).docx [file pone.0273849.s003.docx]

Reply to reviewer(s) Comments

Predicting the Intention to Adopt Wearable Payment Devices in China: The Use of Hybrid SEM-Neural Network Approach

PONE-D-22-02148

**Journal Requirements:**

Please include the following items when submitting your revised manuscript:

1. A rebuttal letter that responds to each point raised by the academic editor and reviewer(s). You should upload this letter as a separate file labeled 'Response to Reviewers'.

**Author Reply:** Thank you. Checked and prepared accordingly

2. A marked-up copy of your manuscript that highlights changes made to the original version. You should upload this as a separate file labeled 'Revised Manuscript with Track Changes'.

**Author Reply:** Thank you. Checked and prepared accordingly

3. An unmarked version of your revised paper without tracked changes. You should upload this as a separate file labeled 'Manuscript'.

**Author Reply:** Thank you. Checked and prepared accordingly

4. If you would like to make changes to your financial disclosure, please include your updated statement in your cover letter. Guidelines for resubmitting your figure files are available below the reviewer comments at the end of this letter.

**Author Reply:** Thank you. Checked and amended accordingly.

5. Please ensure that your manuscript meets PLOS ONE's style requirements, including those for file naming. The PLOS ONE style templates can be found at

<https://journals.plos.org/plosone/s/file?id=wjVg/PLOSOne_formatting_sample_main_body.pdf> and

<https://journals.plos.org/plosone/s/file?id=ba62/PLOSOne_formatting_sample_title_authors_affiliations.pdf>

**Author Reply:** Thank you. Checked and amended accordingly.

6. Please note that PLOS ONE has specific guidelines on code sharing for submissions in which author-generated code underpins the findings in the manuscript. In these cases, all author-generated code must be made available without restrictions upon publication of the work. Please review our guidelines at <https://journals.plos.org/plosone/s/materials-and-software-sharing#loc-sharing-code> and ensure that your code is shared in a way that follows best practice and facilitates reproducibility and reuse.

**Author Reply:** Thank you. No code sharing.

7. Please provide additional details regarding participant consent. In the ethics statement in the Methods and online submission information, please ensure that you have specified what type you obtained (for instance, written or verbal, and if verbal, how it was documented and witnessed). If your study included minors, state whether you obtained consent from parents or guardians. If the need for consent was waived by the ethics committee, please include this information.

**Author Reply:** Thank you. Checked and amended accordingly.

## Under Research Methodology Section (Population and Sample – Sub Section)

Local ethics committee (Universiti Malaysia Kelantan, Malaysia) ruled that no formal ethics approval was required in this particular case because this research did not collect any medical information, there was no known risk involved, did not intend to publish anyone’s personal information, and did not collect data from underaged respondents. This study has been performed in accordance with the Declaration of Helsinki. Informed consent for participation was obtained from respondents who participated in the survey. The respondents who participated in the survey online (using google form) were asked to read the ethical statement posted on the top of the form (*There is no compensation for responding nor is there any known risk. In order to ensure that all information will remain confidential, please do not include your name. Participation is strictly voluntary and you may refuse to participate at any time*) and proceed only if they agree. No data was collected from anyone under 18 years old.

8. Please ensure that you refer to Figure 1 and 2 in your text as, if accepted, production will need this reference to link the reader to the figure.

**Author Reply:** Thank you. Checked and amended accordingly.

Figure 2 represents the ANN model, and figure 3 offers the predicted values by observation.

9. Please include captions for your Supporting Information files at the end of your manuscript, and update any in-text citations to match accordingly. Please see our Supporting Information guidelines for more information: <http://journals.plos.org/plosone/s/supporting-information>.

**Author Reply:** Thank you. Checked and amended accordingly.

**Editorial Comments:**

Thank you for submitting your manuscript to PLOS ONE. After careful consideration, we feel that it has merit but does not fully meet PLOS ONE’s publication criteria as it currently stands. Therefore, we invite you to submit a revised version of the manuscript that addresses the points raised during the review process.

**Ed. Comment 1:** present the model before the hypotheses development;

**Authors Reply:** Research model presented in the study.

**Amendment in the manuscript:**

**Fig 1.** Research Framework

**Ed. Comment 2:** rework on the correlation values of constructs in Table 3

**Authors Reply:** Thank you for the comments. We reported the findings in Table 5. That offer the findings of the Fornell-Larcker criterion to measure constructs' discriminant validity by: (1) the square root of each construct's AVE is higher than its correlation with another construct, and (2) each item loads highest on its associated construct (as presented in Table 6). As the construct's AVE is higher than its correlation with another construct, discriminant validity has been established (Relevant reference: <https://link.springer.com/article/10.1007/s11747-014-0403-8>).

**Ed. Comment 3:** expand the section "Theoretical implications"

**Authors Reply:** Theoretical implication section was improved and extended.

## Amendment in the manuscript:

## Theoretical Implications

This study developed a more sophisticated framework on a well-known theoretical model (e.g., UTAUT2) to better explain the main factors contributing to the formation of an intention to adopt WPD. The study model was extended with two impelling variables (e.g., perceived trust and lifestyle compatibility), which had not been examined in previous studies on contactless payment adoption in China. Another important contribution to the existing literature is the implementation of the two-step analytical method (SEM-ANN), which was shown to be limited in the earlier studies of contactless payments in China that adopted UTAUT2. ANN verified the high prediction accuracy of data fitness through the emphasis on the effects of the most important variables (PT, FC, and PE). The dual analytical strategy offered consistent findings and suggested that more efforts must be undertaken to extend the UTAUT model to explore the technology adoption. The ANN findings suggest that the four prime factors contributing to the formation of an intention to adopt WPDs are perceived trust, lifestyle compatibility, hedonic motivation, and effort expectancy, respectively.

**Ed. Comment 4:** clarify the influence of PE on the intention

**Authors Reply:** Correction made to clarify the study findings for PE on the intention to adopt WPDs.

**Amendment in the manuscript:**

Based on the analysis, the study suggests that the PE for WPDs was found to have a positive and statistically significant impact on the users’ intention to use WPD. The result was consistent with the majority of previous research works in the areas of m-payments (Abrahão et al., 2016; Liébana et al., 2021; Hussain et al., 2019), e-wallet (Yang et al., 2021; Aji et al., 2021), m-wallet (Chawla & Joshi, 2019), m-banking (Alalwan et al., 2017), and wearable payment devices (Lee et al., 2020; Rabaa'i et al., 2021). Contactless payment systems have been widely accepted in China due to the consumers’ belief that contactless payments are perceived as having the performance expectancy to deliver the best services to the users.

**Ed. Comment 5:** include in the section "Discussion" the answer to the three research questions stated in the Introduction;

**Authors Reply:** Corrections made in the introduction and discussion section.

**Amendment in the manuscript:**

*In the introduction section*

The study employed the UTAUT2 components to assess the theory's applicability to the WPD in the Chinese context, given that it is a widely adopted theory to build an understanding of the external and internal drivers of any new technology adoption intention (Koenig et al., 2015). Based on the theoretical analysis, the current study aims to answer the following research questions: (a) what are the critical factors that influence the intention to adopt WPD among Chinese consumers? (b) are the UTAUT2 components sufficient for demonstrating that the influencing factors of adoption intention of WPD or external constructs, such as lifestyle compatibility and perceived trust, are also relevant antecedents?

*In the discussion section*

The current work aims to explore the critical factors that form the intention to use the WPDs. The factors taken from UTAUT2 with additional factors of lifestyle compatibility and perceived trust added to the current study research model. Secondly, we aim to evaluate the UTAUT2 factors explaining the intention to adopt the WPDs with the two additional factors of lifestyle compatibility and perception of trust. Based on the analysis, the study suggests that the PE for WPDs was found to have a positive and statistically significant impact on the users’ intention to use WPD. The result was consistent with the majority of previous research works in the areas of m-payments (Abrahão et al., 2016; Liébana et al., 2021; Hussain et al., 2019), e-wallet (Yang et al., 2021; Aji et al., 2021), m-wallet (Chawla & Joshi, 2019), m-banking (Alalwan et al., 2017), and wearable payment devices (Lee et al., 2020; Rabaa'i et al., 2021). Contactless payment systems have been widely accepted in China due to the consumers’ belief that contactless payments are perceived as having the performance expectancy to deliver the best services to the users. Furthermore, EE had a negative and statistically insignificant effect on the intention to use WPD, indicating that the findings were inconsistent with earlier research (Lee et al., 2021; Lin et al., 2020; Koenig et al., 2015; Oliveira et al., 2016; Chawla & Joshi, 2019). However, because Chinese customers are already accustomed to various sorts of contactless payment systems, it is quite possible that they will overlook the additional effort required to use WPDs.

**Ed. Comment 6:** add a new section on the limitations of the study and future developments;

**Authors Reply:** Necessary amendment made in the manuscript.

**Amendment in the manuscript:**

**Study limitations and Future research options**

However, three pertinent limitations were found in this study. Firstly, the use of cross-sectional design restricted the controllability of unobserved heterogeneity, preventing a strong foundation for proving causality. Therefore, future longitudinal research was suggested to allow variable arrangements to be designed, created, and measured more efficiently with the use of data acquired over a long period of time. Secondly, while the current findings offered a better match in the use of two more variables (LC and PT), other important aspects such as innovativeness, risk, and brand image were overlooked despite their potential to provide a more thorough explanation of the adoption of WPD. By extending the UTAUT2 in the context of Chinese consumers’ intention to use WPD, this study has offered prospects for academics and researchers to conduct future research that includes these components. Considering that UTAUT2 was used to examine behavioural intention, the study scope was broadened to include other behavioural intention theories (e.g., Diffusion of Innovation, Theory of Planned Behaviour) to study the WPD innovation adoption in China. Lastly, the study focused on young people in China who are financially stable and advanced users of technology. Thus, future studies were suggested to examine the model focusing on various demographic populations from a cross-country perspective, which comprises diverse cultural, environmental, economic, and technological circumstances, to explore the behavioural intention of adopting WPD.

**Ed. Comment 7:** include a table presenting the items adopted for constructs' measurement;

**Reply**: survey instrument provided in APPENDIX 1.

**Amendment in the manuscript:**

**APPENDIX 1.** Survey Instrument

| **Code** | **Items** |
| --- | --- |
| PE-1 | Wearable payment device is a useful tool for conducting transactions |
| PE-2 | Wearable payment devices enable me to conduct transactions easily |
| PE-3 | I can conduct transactions faster on wearable payment devices as compared to cash transactions |
| PE-4 | I find wearable payment devices useful in my daily life |
| PE-5 | Using wearable payment devices improves the quality of my daily life |
| PE-6 | Using wearable payment devices helps accomplish things more quickly |
| EE-1 | It would be easy for me to understand the operation of wearable payment devices |
| EE-2 | It is easy for me to become skillful at using wearable payment devices |
| EE-3 | I conduct transactions through mobile devices because many people are doing so |
| EE-4 | Learning how to use wearable payment devices is easy for me |
| EE-5 | I find conducting transactions through devices is convenient for me |
| SI-1 | People who are important to me think that I must use wearable payment devices |
| SI-2 | People who influence my behavior think that I should use wearable payment devices |
| SI-3 | People in my society who use wearable payment devices have more prestigious than those who do not |
| SI-4 | People whose opinions are valuable to me would prefer that I use wearable payment devices |
| SI-5 | People whose opinions I valued preferred that I use this wearable payment devices |
| FC-1 | I have the necessary knowledge to use wearable payment devices |
| FC-2 | Wearable payment devices are compatible with other devices that I use |
| FC-3 | I have the resources necessary to use wearable payment devices |
| FC-4 | Guidance will be available to me in the use of wearable payment devices |
| FC-5 | A specific person (or group) is available for assistance with the difficulties |
| HM-1 | Using wearable payment devices is fun |
| HM-2 | Using wearable payment devices is enjoyable |
| HM-3 | Using wearable payment devices is exciting |
| HM-4 | Using wearable payment devices is delightful |
| HM-5 | Using wearable payment devices is entertaining |
| LC-1 | Using wearable payment devices is compatible with all aspects of my lifestyle |
| LC-2 | Using wearable payment devices is completely compatible with my current situation |
| LC-3 | Using wearable payment devices fits into my lifestyle |
| LC-4 | Using wearable payment services fits well with the way I like to make the bills payment |
| LC-5 | Using wearable payment services instead of alternative modes of payment (e.g. credit card and cash) |
| PT-1 | I believe wearable payment is trustworthy |
| PT-2 | I believe wearable payment keeps customers' best interests in mind |
| PT-3 | I believe wearable payment keeps its promises and commitments |
| PT-4 | I believe wearable payment is reliable |
| PT-5 | Trust and enjoyment become the dominant drivers of wearable payment that offset the risk perceptions |
| IWPD-1 | I intend to use wearable technology at every opportunity in the future |
| IWPD-2 | I plan to increase my use of wearable technology in the future |
| IWPD-3 | It is likely that I will continue using wearable payment devices in future |
| IWPD-4 | Given that I have a smartphone capable of accessing internet, I will continue using wearable payment devices |
| IWPD-5 | I will continue using wearable payment systems in future |
| IWPD-6 | I intend to use wearable devices for planning my payment activities |

**Ed. Comment 8:** add the results of common methods variance;

**Reply:** The assessment of common method variance is reported in the manuscript.

**Amendment in the manuscript:**

**Common Method Variance (CMV)**

In this study, the single factor accounted for 40.281% (below the recommended threshold of 50%), which approved the inconsequential influence of CMV (Podsakoff, Mackenize, & Podsakoff, 2012). CMV evaluated the current study by testing the full collinearity of all constructs (Kock, 2015). All constructs regressed on the common variable. The recorded values of the variance inflation factor (as presented in Table 2) were less than 3.3, suggesting the absence of bias from the single-source data (Kock, 2015).

**Table 2.** Full Collinearity Test

|  | PE | EE | SI | FC | HM | LC | PT | IWPD |  |
| --- | --- | --- | --- | --- | --- | --- | --- | --- | --- |
| Variance inflation factor (VIF) Values | 2.601 | 2.515 | 2.117 | 2.720 | 1.438 | 1.644 | 1.675 | 3.078 |  |

**Note:** “PE: Performance Expectancy; EE: Effort Expectancy; SI: Social Influence; FC: Facilitating Conditions; HM: Hedonic Motivation; LC: Lifestyle Compatibility; PT: Perceived Trust; IWPD: Intention to Adopt WPDs

**Source:** authors’ data analysis.”

**Ed. Comment 9:** clarify in the section "Literature review" that the UTAUT2 constructs "price value" and "habit" were NOT considered in this study;

**Authors Reply:** Clarification was offered to justify the non-inclusion of price value and habit variable in the current study model.

**Amendment in the manuscript:**

The majority of earlier research works (Morosan et al., 2016; Hussain et al., 2019; Gupta and Arora, 2019; Oliveira et al., 2016) found that price value was not a significant predictor of adoption intention in the context of an upgraded technology with contactless payments. Following the literature results and judgments, price value was taken into account in this study. Customers should acquire high experience in adopting technology to investigate the function of habit (Alalwan et al., 2017). Furthermore, a relatively new technology that has not acquired significant market acceptance may be unable to establish a habit (Oliveira et al., 2016; Lin et al., 2020). Notably, many of the participants in this study were potential users who were yet to use WPD or use it. As a result, the researcher assumed not to include the price value and habit in the current study model.

**Ed. Comment 10:** include in the section "Discussion" some considerations about the possible effects of the sample composition on the results;

**Authors Reply:** We agree with the editor about the possible effect of sample demographics on the intention to adopt the WPDs. Therefore, we reported the current study sample demographics as a limitation.

**Amendment in the manuscript:**

Lastly, the study focused on young people in China who are financially stable and advanced users of technology. Thus, future studies were suggested to examine the model focusing on various demographic populations from a cross-country perspective, which comprises diverse cultural, environmental, economic, and technological circumstances to explore the behavioural intention of adopting WPD.

**Ed. Comment 11:** revise the manuscript correcting typos (e.g., at the end of page 13 "Fndings").

**Authors Reply:** We thoroughly checked and necessary corrections made in the manuscript.

**Amendment in the manuscript:**

Findings indicated that LC

**Ed. Comment 12:** UTAUT2 construct "price value" was not considered in this study. As a consequence the sentence "Following the aforementioned literature results and judgments, price value was taken into account in this study" (Section "Literature review", page 4) should be modified. Also the UTAUT2 construct "habit" was not included in the present study. On this respect the sentence "As a result, investigating the impact of habit was challenging, which led to the decision to adopt WPD" should be clarified.

**Authors Reply:** Correction made in the section.

**Amendment in the manuscript:**

The majority of earlier research works (Morosan et al., 2016; Hussain et al., 2019; Gupta and Arora, 2019; Oliveira et al., 2016) found that price value was not a significant predictor of adoption intention in the context of an upgraded technology with contactless payments. Following the literature results and judgments, price value was not taken into account in this study. Customers should acquire high experience in adopting technology to investigate the function of habit (Alalwan et al., 2017). Furthermore, a relatively new technology that has not acquired significant market acceptance may be unable to establish a habit (Oliveira et al., 2016; Lin et al., 2020). Notably, many of the participants in this study were potential users who were yet to use WPD or use it. As a result, the researcher assumed not to include the price value and habit in the current study model.

**Ed. Comment 12:** Some considerations about the possible effects of the sample composition on the results should be included in the discussion. The results could be influenced by the composition of the sample e. g. the influence of FC on users’ intention to adopt WPD is not significant in this study with a sample composed by young people. Probably a different result could be obtained adopting a sample composed by people of a different age range.

**Authors Reply:** Correction made in the statement.

**Amendment in the manuscript:**

Lastly, the study focused on young people in China who are financially stable and advanced users of technology. Thus, future studies were suggested to examine the model focusing on various demographic populations from a cross-country perspective, which comprises diverse cultural, environmental, economic, and technological circumstances to explore the behavioural intention of adopting WPD.

**Reviewers' comments:**

**Reviewer #1:**

**Rv. Comment 13:** Please present the model before the hypotheses development.

**Authors Reply:** We added the research model in Figure 1.

**Amendment in the manuscript:**

**Fig 1.** Research Framework

**Rv. Comment 14:** You need to rework on the correlation values of constructs in Table 3. Some of them are very high. In fact, anything more than 0.8 is alarming and indicates that the two constructs are similar in nature. You have many of them more than .800. Please fix this issue.

**Authors Reply:** Thank you for the comments. We reported the findings in Table 5. That offer the findings of the Fornell-Larcker criterion to measure constructs' discriminant validity by (1) the square root of each construct's AVE is higher than its correlation with another construct, and (2) each item loads highest on its associated construct (as presented in Table 6). As the construct's AVE is higher than its correlation with another construct, discriminant validity has been established (Relevant reference: <https://link.springer.com/article/10.1007/s11747-014-0403-8>).

**Rv. Comment 15:** The model says that PE in non-significant on intentions. Why? It's a matter of great concern as if wearable watches are not significant then why this all the hard work for?

**Authors Reply:** As we were asked to check CMB, we rechecked the data and found many outliers, i.e. reply to that all questions were same, so we removed the outliers and re-analyzed. New findings show that PE and FC have a positive effect on IWPD.

**Amendment in the manuscript:**

Based on the analysis, the study suggests that the PE for WPDs was found to have a positive and statistically significant impact on the users’ intention to use WPD. The result was consistent with the majority of previous research works in the areas of m-payments (Abrahão et al., 2016; Liébana et al., 2021; Hussain et al., 2019), e-wallet (Yang et al., 2021; Aji et al., 2021), m-wallet (Chawla & Joshi, 2019), m-banking (Alalwan et al., 2017), and wearable payment devices (Lee et al., 2020; Rabaa'i et al., 2021).

**Rv. Comment 16:** Theoretical implications are weak. Please strengthen it further.

**Authors Reply:** The theoretical implication section improved.

**Amendment in the manuscript:**

## Theoretical Implications

This study developed a more sophisticated framework on a well-known theoretical model (e.g., UTAUT2) to better explain the main factors contributing to the formation of an intention to adopt WPD. The study model was extended with two impelling variables (e.g., perceived trust and lifestyle compatibility), which were not examined in previous studies on contactless payment adoption in China. Another vital contribution to the existing literature is the implementation of the two-step analytical method (SEM-ANN), which was shown to be limited in the earlier studies of contactless payments in China that adopted UTAUT2. ANN verified the high prediction accuracy of data fitness through the emphasis on the effects of the most important variables (PT, LC, and HM). The dual analytical strategy offered consistent findings and suggested that more efforts must be undertaken to extend the UTAUT model to explore the technology adoption. The ANN findings suggest that the four prime factors contributing to the formation of an intention to adopt WPDs are perceived trust, lifestyle compatibility, hedonic motivation, and effort expectancy, respectively.

**Rv. Comment 17:** Please keep a section on limitations and future research separately.

**Authors Reply:** Correction made as suggested by the reviewer.

**Amendment in the manuscript:**

**Study limitations and Future research options**

However, three pertinent limitations were found in this study. Firstly, the use of cross-sectional design restricted the controllability of unobserved heterogeneity, preventing a strong foundation for proving causality. Therefore, future longitudinal research was suggested to allow variable arrangements to be designed, created, and measured more efficiently with the use of data acquired over a long period of time. Secondly, while the current findings offered a better match in the use of two more variables (LC and PT), other important aspects such as innovativeness, risk, and brand image were overlooked despite their potential to provide a more thorough explanation of the adoption of WPD. By extending the UTAUT2 in the context of Chinese consumers’ intention to use WPD, this study has offered prospects for academics and researchers to conduct future research that includes these components. Considering that UTAUT2 was used to examine behavioural intention, the study scope was broadened to include other behavioural intention theories (e.g., Diffusion of Innovation, Theory of Planned Behaviour) to study the WPD innovation adoption in China. Lastly, the study focused on young people in China who are financially stable and advanced users of technology. Thus, future studies were suggested to examine the model focusing on various demographic populations from a cross-country perspective, which comprises diverse cultural, environmental, economic, and technological circumstances to explore the behavioural intention of adopting WPD.

**Rv. Comment 18:** You have mentioned that PE has a minor impact on intentions in discussion whereas it is found non-significant. This is not acceptable. Please provide the reasons for this non-significant impact. The rationale provided for it is not logical.

**Authors Reply:** As we were asked to check CMB, we rechecked the data and found many outliers. A large portion of respondents' responses to all questions was the same, and we removed the outliers and re-analyzed. New findings show that PE and FC have a positive effect on IWPD.

**Amendment in the manuscript:**

Based on the analysis, the study suggests that the PE for WPDs was found to have a positive and statistically significant impact on the users’ intention to use WPD. The result was consistent with the majority of previous research works in the areas of m-payments (Abrahão et al., 2016; Liébana et al., 2021; Hussain et al., 2019), e-wallet (Yang et al., 2021; Aji et al., 2021), m-wallet (Chawla & Joshi, 2019), m-banking (Alalwan et al., 2017), and wearable payment devices (Lee et al., 2020; Rabaa'i et al., 2021).

**Reviewer #2:**

**Rv. Comment 19:** In the discussion section, it should answer the three research questions stated in the introduction section. For example, the mediating effects of behavioural intention on the relationship between the predictors and actual adoption.

**Reply:** Necessary correction made in the introduction and discussion section.

**Amendment in the manuscript:**

*In the introduction section*

The study employed the UTAUT2 components to assess the theory's applicability to the WPD in the Chinese context, given that it is a widely adopted theory to build an understanding of the external and internal drivers of any new technology adoption intention (Koenig et al., 2015). Based on the theoretical analysis, the current study aims to answer the following research questions: (a) what are the critical factors influencing the intention to adopt WPD among Chinese consumers? (b) are the UTAUT2 components sufficient for demonstrating that the influencing factors of adoption intention of WPD or external constructs, such as lifestyle compatibility and perceived trust, are also relevant antecedents?

*In the discussion section*

The current work aims to explore the critical factors that form the intention to use the WPDs. The factors taken from UTAUT2 with additional factors of lifestyle compatibility and perceived trust added to the current study research model. Secondly, we aim to evaluate the UTAUT2 factors explaining the intention to adopt the WPDs with the two additional factors of lifestyle compatibility and perception of trust. Based on the analysis, the study suggests that the PE for WPDs was found to have a positive and statistically significant impact on the users’ intention to use WPD. The result was consistent with the majority of previous research works in the areas of m-payments (Abrahão et al., 2016; Liébana et al., 2021; Hussain et al., 2019), e-wallet (Yang et al., 2021; Aji et al., 2021), m-wallet (Chawla & Joshi, 2019), m-banking (Alalwan et al., 2017), and wearable payment devices (Lee et al., 2020; Rabaa'i et al., 2021). Contactless payment systems have been widely accepted in China due to the consumers’ belief that contactless payments are perceived as having the performance expectancy to deliver the best services to the users. Furthermore, EE had a negative and statistically insignificant effect on the intention to use WPD, indicating that the findings were inconsistent with earlier research (Lee et al., 2021; Lin et al., 2020; Koenig et al., 2015; Oliveira et al., 2016; Chawla & Joshi, 2019). However, because Chinese customers are already accustomed to various sorts of contactless payment systems, it is quite possible that they will overlook the additional effort required to use WPDs.

**Rv. Comment 20:** Before indicating validity and descriptive analysis, I think that the authors need to uncover the measurement items. However, I cannot see any table that indicating the measurements.

**Reply:** Result provided in Table 1.

**Amendment in the manuscript:**

**Table 1.** Measurements

| Factors | No of Items | Minimum | Maximum | Mean | Std. Deviation |
| --- | --- | --- | --- | --- | --- |
| PE | 6 | 1.330 | 5.000 | 3.968 | 0.724 |
| EE | 5 | 1.000 | 5.000 | 3.966 | 0.706 |
| SI | 5 | 1.000 | 5.000 | 3.370 | 1.093 |
| FC | 5 | 1.000 | 5.000 | 3.848 | 0.743 |
| HM | 5 | 1.000 | 5.000 | 3.885 | 0.761 |
| LC | 5 | 1.400 | 5.000 | 3.746 | 0.819 |
| PT | 5 | 1.000 | 5.000 | 3.573 | 0.778 |
| WPD | 6 | 1.000 | 5.000 | 3.805 | 0.812 |

**Note:** “PE: Performance Expectancy; EE: Effort Expectancy; SI: Social Influence; FC: Facilitating Conditions; HM: Hedonic Motivation; LC: Lifestyle Compatibility; PT: Perceived Trust; IWPD: Intention to Adopt WPDs

**Source:** authors’ data analysis.

**Rv. Comment 21:** Please add the results of common methods variance.

**Reply:** The assessment of common method variance is included in the manuscript.

**Amendment in the manuscript**

**Common Method Variance (CMV)**

In this study, the single factor accounted for 40.281% (below the recommended threshold of 50%), which approved the inconsequential influence of CMV (Podsakoff, Mackenize, & Podsakoff, 2012). CMV evaluated the current study by testing the full collinearity of all constructs (Kock, 2015). All constructs regressed on the common variable. The recorded values of the variance inflation factor (as presented in Table 2) were less than 3.3, suggesting the absence of bias from the single-source data (Kock, 2015).

**Table 2.** Full Collinearity Test

|  | PE | EE | SI | FC | HM | LC | PT | IWPD |  |
| --- | --- | --- | --- | --- | --- | --- | --- | --- | --- |
| Variance inflation factor (VIF) Values | 2.601 | 2.515 | 2.117 | 2.720 | 1.438 | 1.644 | 1.675 | 3.078 |  |

**Note:** “PE: Performance Expectancy; EE: Effort Expectancy; SI: Social Influence; FC: Facilitating Conditions; HM: Hedonic Motivation; LC: Lifestyle Compatibility; PT: Perceived Trust; IWPD: Intention to Adopt WPDs

**Source:** authors’ data analysis.”

**Rv. Comment 22:** Why the author not applied PLSPredict in PLS-SEM?

**Authors Reply:** We utilized dual analytical techniques to build the consistent and valid study results. PLSpredict is good for testing the PLS-SEM model and LM (Linear Model) validity with errors produced by two kinds of model. However, we think that it was not our objective to utilize the PLSpredict analysis for the current work.
