# Supplementary material for: Predicting the intention to adopt wearable payment devices in China: The use of hybrid SEM-Neural network approach
Source: PLoS One. 2022 Aug 30;17(8):e0273849. doi: 10.1371/journal.pone.0273849 (PMC9426926; doi:10.1371/journal.pone.0273849)
Supplement: Attachment — Submitted filename: Reply to Reviewers Comm - PONE-D-22-02148R1.docx [file pone.0273849.s004.docx]

**Reply to Reviewer(s) Comments**

PONE-D-22-02148R1

**Comments to the Author**

1. If the authors have adequately addressed your comments raised in a previous round of review and you feel that this manuscript is now acceptable for publication, you may indicate that here to bypass the “Comments to the Author” section, enter your conflict of interest statement in the “Confidential to Editor” section, and submit your "Accept" recommendation.

Reviewer #1: (No Response)

Reviewer #2: All comments have been addressed

Reviewer #3: All comments have been addressed

2. Is the manuscript technically sound, and do the data support the conclusions?

The manuscript must describe a technically sound piece of scientific research with data that supports the conclusions. Experiments must have been conducted rigorously, with appropriate controls, replication, and sample sizes. The conclusions must be drawn appropriately based on the data presented.

Reviewer #1: No

Reviewer #2: Yes

Reviewer #3: Yes

3. Has the statistical analysis been performed appropriately and rigorously?

Reviewer #1: Yes

Reviewer #2: Yes

Reviewer #3: Yes

4. Have the authors made all data underlying the findings in their manuscript fully available?

The [PLOS Data policy](http://www.plosone.org/static/policies.action#sharing) requires authors to make all data underlying the findings described in their manuscript fully available without restriction, with rare exception (please refer to the Data Availability Statement in the manuscript PDF file). The data should be provided as part of the manuscript or its supporting information, or deposited to a public repository. For example, in addition to summary statistics, the data points behind means, medians and variance measures should be available. If there are restrictions on publicly sharing data—e.g. participant privacy or use of data from a third party—those must be specified.

Reviewer #1: No

**Authors Reply:** Kindly refer to the data provided as Supporting Information: Data - Predicting the Intention to Adopt WPDs.sav

Reviewer #2: Yes

Reviewer #3: Yes

5. Is the manuscript presented in an intelligible fashion and written in standard English?

PLOS ONE does not copyedit accepted manuscripts, so the language in submitted articles must be clear, correct, and unambiguous. Any typographical or grammatical errors should be corrected at revision, so please note any specific errors here.

Reviewer #1: Yes

Reviewer #2: Yes

Reviewer #3: Yes

6. Review Comments to the Author

Please use the space provided to explain your answers to the questions above. You may also include additional comments for the author, including concerns about dual publication, research ethics, or publication ethics. (Please upload your review as an attachment if it exceeds 20,000 characters)

Reviewer #1: This study aims to investigate the effects of core constructs of UTAUT along with lifestyle compatibility and perceived trust to understand consumer intention to adopt WPD. The data were collected from 298 consumers in China. The PLS-SEM and ANN analysis were conducted to analyse the data. The authors have come up with some interesting findings. However, the paper has a number of issues as well:

1. Inadequate research analysing the behavioral elements influencing adoption of WPD is not a compelling reason for undertaking this research. The research gap should be found out based on the gaps from the existing literature.

**Authors Reply**: Thank you for your suggestions prof. We amended our the motivation and research gap.

**Amended Manuscript** (Added):

Despite the usefulness and benefits of adopting wearable payment devices, there is a lack of study on wearable payment device adoption behaviour considering they are considerably in the early stages of commercialization. Interestingly, research in the context of China on m-payment usage (Cao et al., 2018; Chen et al., 2019; Huang et al., 2020) and wearable technology adoption (Chuah et al., 2016) has sought to uncover the factors driving technological product and service adoption. Furthermore, while m-payment and WPD serve the same purposes, there are significant technological and procedural differences that distinguish WPD from m-payment (Lee et al., 2020). Additionally, an individual’s behavioural factors toward different products and services are totally subjective, and thus their adoption behaviour is likely to be different for each. So, understanding of m-payment of adoption behaviour, it may not be useful for product innovators and shoppers of WPD. Identifying research gaps in wearable payment device adoption behaviour, the current study investigates factors impacting WPD adoption in China.

2. There is no need of bringing the selection of what constructs to be used in the proposed model under introduction section.

**Authors Reply**: Thank you for your suggestions prof. We removed the irrelevant part as recommended (discussion about constructs)

3. The data is very skewed and hence does not provide the full understanding of broader range of customers of various age groups. It generalisation is very limited. There is no meaning of understanding the adoption of any such technology for young generation as they are quite well-versed with these technologies anyway. You need to provide convincing rationale for undertaking this research. There is no novelty of doing it as everyone knows that such new technologies are well adopted within the younger generation anyway.

**Authors Reply**: Thank you for the comment. I understand your frustration, research in social science often feels like ‘mentioning the obvious’. If we criticise like this, we can criticise almost all the papers published in all journals.

Saying that, I am not sure how to reply to this comment or what to improve based on this comment as there is nothing specific.

4. There is nothing new being reported based on the analysis. EE is coming non-significant as usual so are other variables as significant as well.

**Authors Reply**: Again, there is nothing specific that we can do based on the comment above.

5. Discussion does not provide anything new from findings. Just saying that the findings are in line with the previous studies clearly saying that there is nothing new in the paper.

**Authors Reply**: We have highlighted PT and LC as new findings of the our study in the context WPD.

**Amended Manuscript** (Highlighted BLUE):

Typical model sensitivity evaluations include ‘one-at-a-time’ simulations that individually evaluate the impact of each independent variable while overlooking the interactions with other independent variables (Beres & Hawkins, 2001). In ANN, sensitivity analysis enables the evaluation of input variables in terms of the significance of their impact on the output variable and the identification of factors that could be omitted without compromising network quality and the critical key factors (Mrzygłód, 2020). The mean importance of ANN sensitivity analysis (Table 9) in this study dataset indicated that PT was the most important variable, followed by LC and HM. Figure 2 represents the ANN model, and Figure 3 offers the predicted values by observation.

6. This is the reason why theoretical and practical implications are quite limited.

**Authors Reply**: Thank you. We have extended implications.

**Amended Manuscript** (Highlighted BLUE):

# Implication

## *Theoretical Implications*

The main objective of the current study is examining factor affecting WPD adoption behaviour in China. In doing so, this study developed a more sophisticated framework based on a well-known UTAUT2) of Venkatesh et al. (2012) to capture contributing factors to the formation of the intention to adopt WPD. Our model capture 68% of IWPD which confirms the validity of the UTAUT2 in context of WPD, and the findings highlighted UTUAT2 factors are important in formation of IWPD. Thus, it extends the literature of contactless payment. Furthermore, as mentioned earlier, wearable payment devices are currently in the early stages of commercialization and there is lack of studies in this context, our study contributes to the model and theoretical development in WPD adoption behaviour. Moreover, unlike previous studies on contactless payment adoption, this study included perceived trust and lifestyle compatibility as influencing factors of IWPD, which were never explored in the context of contactless payment especially wearable device. The significant influences of perceived trust and lifestyle compatibility adds new evidence to the literature in contactless payment.

In addition to SEM-PLS, the current study employed a two-step analytical approach (SEM-ANN) to confirm factors that contribute to the formulation of an IWPD. This ANN validated the excellent predictive accuracy of data fitness by focusing on the impacts of the most important variables. Such empirical estimation is still limited in previous research of contactless payments in China that used UTAUT2. The current study is the first attempt in using an integrated ANN model to observe the predictors of WPD adoption variables at the individual level. In our cases, the dual analytical strategy offered consistent findings and suggested that more efforts be undertaken to extend the UTAUT model to explore technology adoption. The ANN findings suggested that the four prime factors contributing to the formation of an intention to adopt WPDs are PT, FC and PE, respectively. Hence, it offers important contribution to the literature of contactless payment. Finally, the current study explored in the context of the China market thus improving literature of the China market and offering literature to compare with other countries.

*Practical Implications*

The primary goal of this study is to identify and validate a set of attributes that enhance the behavioural intention to use WPD systems in China.  The current study offers a few practical implications for product innovators, financial institutions, and shoppers. The current study shows that PE, EE, FC, HM, LC, and PT have a substantial impact on WPD adoption. Such knowledge can assist financial institutions and device manufacturers in developing new plans and marketing methods to persuade WPD adopters. For example, relating to performance expectations, wearable payment device manufacturers and financial institutions should improve the functionalities and features of their devices, where they may focus on ensuring availability, performance, effectiveness, and efficiency, including 24*7 services and customer support. Referring to effort efficiency, WPD should be easy-to-use with an adaptable user interface and a high-quality product experience. The significance of facilitating conditions suggests that WPD manufacturers should include a practical benefit and full instructions to be effectively conveyed to the consumers via marketing and social media to spread information and the advantages of WPD among users.

Furthermore, the significance of lifestyles implies that the adoption and usage of different models of WPD is likely to change in China with changing lifestyles and fashion trends because wearable device are currently deemed as fashion accessory. In market like China, owning a high-end technology device symbolize the high social status (Gao et al. 2014). Hence, in an attempt to hold and increase market share in the WPD industry, device manufacturers should architect and develop new products with continuous market assessment where they need to assess how their products suit customers’ lifestyles and current fashion trends. Additionally, device makers must evaluate the needs and lifestyles of customers in various segments in order to design and manufacture the products that best suit those customers. In advertising, WPD manufacturers and service providers should consider the living standards and lifestyle patterns of their target customer segments, where they may hire a fashion icon as their brand ambassador to promote products.

Moreover, trust has always been a critical key factor in financial services and technological products. The relevance of trust and security-related concerns is further highlighted by the discovery that perceived trust is a crucial determinant of the intention to use WPD. Hence, manufacturers and service providers should determine what customers perceive as trust-related risks associated with WPD usage in order to make WPD more trustworthy (Liébana et al., 2021). Therefore, in the first place, manufacturers should establish the safest possible infrastructure and environment for WPD services. Then, WPD providers should disclose information detailing data privacy rules, end-to-end encryption, biometric authentication capabilities (e.g., fingerprint readers), secured networks, and sensor-based procedures, which make customers feel trustworthy and protected. Additionally, they need to ensure that their WPD is error free in terms of software functioning. On the other hand, financial institutions, as a service provider, have to provide data security, service accessibility, platform security for wearable device payments, and user assurance that they can execute a variety of financial transactions efficiently and safely utilizing their wearable payment devices. Financial institutions may make use of a number of sophisticated encryption and authentication techniques to increase security. All these measures will ultimately increase trust of users towards device and servicer providers.

Finally, the findings would assist the government and key official policymakers in the governing bodies in improving the policies to enhance wide acceptance of WPD and accomplish the goal of developing a cashless society.

Reviewer #2: (No Response)

Reviewer #3: The statistical analysis in the current version of the paper is well done and described. I have no comments for the authors and I suggest accepting the paper in its current version.

**Authors Reply**: Thank you Prof.
